# Protective Effects of Gallic Acid Against Lead Acetate‐ Induced Toxicity in Mice Ovary: Focus on Apoptosis, Inflammation, and Folliculogenesis

**DOI:** 10.1002/fsn3.70638

**Published:** 2025-07-16

**Authors:** Fatemeh Zahedi, Rasoul Kowsar, Zahra Khodabandeh, Mahintaj Dara, Sanaz Alaee

**Affiliations:** ^1^ Department of Animal Sciences, College of Agriculture Isfahan University of Technology Isfahan Iran; ^2^ Stem Cells Technology Research Center Shiraz University of Medical Sciences Shiraz Iran; ^3^ Infertility Research Centre Shiraz University of Medical Sciences Shiraz Iran; ^4^ Department of Reproductive Biology, School of Advanced Medical Sciences and Technologies Shiraz University of Medical Sciences Shiraz Iran; ^5^ Department of Natural Sciences West Kazakhstan Marat Ospanov Medical University Aktobe Kazakhstan

**Keywords:** antioxidant, apoptosis, gallic acid, inflammation, lead acetate, miRNA

## Abstract

Lead is an environmental pollutant recognized for its toxicity to the reproductive system. This study investigated the effects of gallic acid (GA), a polyphenolic compound found in plants, on the ovaries of mice exposed to lead (Pb) in the form of lead acetate. Forty *Balb*/*c* mice were divided into five groups: control (no treatment), sham (normal saline), GA (75 mg/kg), Pb (30 mg/kg), and GA (75 mg/kg) + Pb (30 mg/kg). Daily treatments were administered for 35 days via oral gavage, after which the ovaries were examined. The mRNA expression of antioxidant, inflammatory, and apoptotic genes as well as two microRNAs (miRs) involved in folliculogenesis, was measured using real‐time reverse transcription polymerase chain reaction (RT‐PCR). The results of RT‐PCR indicated that Pb increased the expression of *miR‐132, Il‐1β* (Interleukin‐1 beta), *Tnf*‐*α* (Tumor necrosis factor alpha), *P53* (Tumor protein 53), *Caspase‐3* (*p* ≤ 0.05), and *Nf‐κB* (Nuclear factor kappa‐light‐chain‐enhancer of activated B cells) compared to the control group significantly (*p* ≤ 0.01). Pb remarkably reduced the expression of *Vegf* (Vascular endothelial growth factor) compared to the sham group (*p* ≤ 0.05). GA elevated the expression of *miR‐125b*, *Gpx* (Glutathione peroxidase), and *Gr* (Glutathione reductase) compared to the control group, while also increasing the expression of *Bcl‐2* (B‐cell lymphoma 2 apoptosis regulator) compared to the sham group, all of them statistically significant (*p* ≤ 0.05). In the (GA + Pb) group, GA increased the expression of *miR‐125b, Bcl‐2,* and *Vegf,* while it decreased the expression of *Caspase‐3* compared to the Pb group significantly (*p* ≤ 0.05). In summary, exposure to lead acetate (Pb) demonstrates detrimental effects on ovarian function by reducing antioxidant capacity and inducing inflammation and apoptosis. In contrast, gallic acid (GA) plays a protective role and mitigates the harmful effects of lead on the ovaries.

## Graphical abstract

Schematic representation of the impact of lead acetate exposure on ovarian tissue. (b) The therapeutic potential of gallic acid in restoring antioxidant balance and mitigating toxicity in female mice.

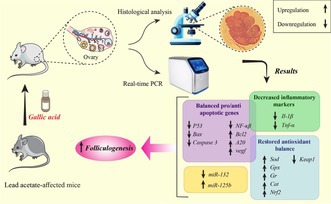

## 1 Introduction

Heavy metals are toxic elements like lead, mercury, and cadmium that persist in the environment. Their accumulation in living organisms can cause severe health issues, including damage to the nervous, reproductive, and immune systems (Jomova et al. 2025). Studies indicate that heavy metals damage the reproductive system in females by impairing ovarian tissue, disrupting follicular development and ovulation, and potentially leading to hormonal imbalances and reduced fertility (Sanaz Alaee 2018; Wrzecinska et al. 2021). Lead (Pb) is a toxic heavy metal that is widely utilized in various industries due to its unique properties, including softness, high malleability, flexibility, low melting point, and resistance to corrosion (Angon et al. 2024). However, exposure to Pb damages various organ systems, including the nervous, hematologic, gastrointestinal, immune, and reproductive systems (Shan et al. 2023). Pb affects the function of ovaries, reducing the number of primordial and primary follicles while increasing the number of atretic follicles (Shan et al. 2023). Pb impacts fertility by creating hormonal imbalances and disrupting the hypothalamic–pituitary–gonadal axis, resulting in decreased production of gonadotropins and sex hormones (Plunk and Richards 2020).

Lead acetate causes the generation of reactive oxygen species (ROS), which disrupt the balance between oxidants and antioxidants in ovarian tissue. This oxidative stress leads to cellular damage to lipids, proteins, and DNA, ultimately affecting ovarian function and causing inflammation (Tumilaar et al. 2024). A study on bovine granulosa cells (GCs) indicated that oxidative stress induced by lead (Pb) affects the proliferation of GCs and increases inflammation and apoptosis (Aglan et al. 2020). In a healthy organism, inflammatory and anti‐inflammatory cytokines are balanced. When inflammatory cytokines such as *Il‐1β* (Interleukin‐1 beta) and *Tnf‐α* (Tumor necrosis factor alpha) bind to specific cellular receptors, they activate signaling proteins like *Nf‐κB* (Nuclear factor kappa‐light‐chain‐enhancer of activated B cells) (Aggeletopoulou et al. 2024). *Nf‐κB* stimulates the expression of *the P53* (Tumor protein 53) gene to maintain the body's homeostasis (Anilkumar and Wright‐Jin 2024). *P53* induces apoptosis (programmed cell death) to prevent damage caused by Pb. The *Bcl‐2* (B‐cell lymphoma 2 apoptosis regulator) gene family plays a significant role in apoptosis (Senturk and Manfredi 2013). *Bax* (Bcl‐2‐associated X protein), an apoptotic protein of this family, creates a pore in the mitochondrial membrane, subsequently allowing *cytochrome C* to exit the mitochondria and enter the cytoplasm. This process activates *caspase* proteases, which lead to protein degradation (Ilani et al. 2018). *Caspase‐3* is the most important factor in cell death (Bolouki et al. 2020). *Bcl‐2*, an anti‐apoptotic protein of the *Bcl‐2* family, maintains the integrity of the mitochondrial membrane (Ilani et al. 2018).

Some other anti‐apoptotic genes are expressed to maintain homeostasis. A20, known as Tnf‐αip3 (Tnf‐α‐induced protein 3), is a signaling protein recognized as an anti‐inflammatory molecule and an inhibitor of Nf‐κB that aids in preventing excessive apoptotic activity (Momtazi et al. 2019). Vegf (vascular endothelial growth factor) is an anti‐apoptotic glycoprotein that specifically binds to receptors present on endothelial cells, promoting the division and proliferation of these cells. Vegf plays a central role in ovarian angiogenesis. Disruption of ovarian angiogenesis may lead to infertility, miscarriage, and other related issues (Pan et al. 2020).

The *Nrf2* (nuclear factor erythroid 2‐related factor 2) signaling pathway acts as a “master regulator” of the cellular antioxidant defense system. *Nrf2*, as an active transcription factor, regulates the expression of antioxidant genes located in the promoter region of antioxidant response elements (*AREs*) (Kanwugu and Glukhareva 2023). Antioxidant enzymes include *Sod* (Superoxide dismutase), *Cat* (Catalase), *Gpx* (Glutathione peroxidase), and *Gr* (Glutathione reductase) (Jomova et al. 2024). Activation of *Nrf2* leads to a coordinated antioxidant and anti‐inflammatory response. In non‐stressed cells, *Keap1* (Kelch‐like ECH‐associated protein 1) causes ubiquitination and degradation of *Nrf2* to prevent the expression of unnecessary genes (Nguyen et al. 2009).

Any substance that enters the body causes epigenetic changes, such as DNA methylation, histone acetylation, and microRNA modulation. MicroRNA (miR) is a 19‐22‐nucleotide RNA that is not translated into protein but regulates gene expression (Agullo et al. 2024). The expression of miRNAs is essential in ovarian functions. The levels of miRNAs change during growth, differentiation, and apoptosis of the ovarian follicles (Kusumaningtyas et al. 2024). A study on mouse ovaries found that *miR‐125b* and *miR‐132* are expressed in antral follicles but not in the secondary follicles (Kusumaningtyas et al. 2024).

Natural ingredients from medicinal plants exhibit anti‐inflammatory, antioxidant, and anti‐apoptotic detoxifying properties (Bjørklund et al. 2024; Monsefi et al. 2015). These compounds help neutralize oxidative stress, reduce cellular damage, and support the repair of reproductive tissues. By enhancing detoxification pathways and modulating hormone regulation, plant‐based ingredients offer a promising, natural approach to safeguarding fertility and counteracting the adverse impacts of toxicants on both male and female reproductive systems (Alaee et al. 2024; Jahromi et al. 2019). Gallic acid (GA) is a naturally occurring phenolic compound widely used in the food, pharmaceutical, cosmetic, and personal hygiene industries due to its antioxidant, antimicrobial, and anti‐inflammatory properties (Akbarzadeh‐Jahromi et al. 2022; Bai et al. 2021).

This study investigated the protective effects of gallic acid (GA) against lead (Pb)‐induced ovarian toxicity in mice, focusing on the impact of GA on oxidative stress (via Nrf2‐Keap1 pathway and antioxidant genes Cat, Gpx, Gr, Sod), inflammation (through A20/TNFAIP3, Il‐1β, Tnf‐α, Nf‐κB), apoptosis (P53, Bax, Caspase‐3, Bcl‐2), angiogenesis (Vegf), and folliculogenesis‐related gene expression, including microRNAs miR‐132 and miR‐125b, in response to lead acetate exposure. The study aimed to determine whether GA could mitigate Pb‐induced ovarian damage by modulating these critical pathways and support the restoration of normal ovarian function.

## 2 Materials and Methods

### 2.1 Animal and Treatments

Forty adult female mice of the *Balb/c* strain, weighing 25–30 g (7 weeks old), were purchased from the animal house of Shiraz University of Medical Sciences, Iran. The ethical committee at Shiraz University of Medical Sciences (IR.SUMS.REC.1400.199) approved all procedures involving animals. This work was conducted according to national and institutional guidelines for the care and use of laboratory animals. Animals were housed in standard cages at 22°C–24°C. They had a 12 h light/12 h dark cycle and had unlimited access to water and food (Pars Dam Co., Tehran, Iran). The humidity was 40%–60%. After 14 days of adaptation, the animals were randomly allocated to five groups (*n* = 8/group): (1) control group (without treatment); (2) sham group (received normal saline); (3) GA group received gallic acid (75 mg/kg) (Sigma‐Aldrich, USA; Cat. No G7384‐25G); (4) Pb group received lead acetate (30 mg/kg) (Merck, Darmstadt, Germany); (5) The GA + Pb group received GA (75 mg/kg) + Pb (30 mg/kg) (gallic acid was treated one hour before lead acetate). The administered doses of gallic acid and lead acetate were selected based on previous studies (Ahmed et al. 2019; Ul Haq Shah et al. 2022; Widawati et al. 2017). All treatments were administered orally (via gavage) daily for 35 days. At the end of the experiment, animals were weighed and anesthetized using Ketamine‐xylazine and sacrificed via cardiac puncture. The left ovaries were kept at −80°C for miRNA and mRNA analysis. The right ovaries were preserved in buffered formalin for simple microscopic observation of ovarian tissue (Duncan et al. 2015).

### 2.2 RNA Isolation, cDNA Synthesis, and Real‐Time RT‐PCR

#### 2.2.1 RNA Isolation

Total RNA was extracted from homogenized ovarian tissue using 1 mL of RNX‐PLUS reagent (Sinaclon, Iran; Cat. No EX6101), following the manufacturer's protocol. Phase separation was performed with 200 μL of chloroform (Chem‐labco, Iran; Cat. No A9135), followed by centrifugation at 12,000 rpm for 15 min. The aqueous phase was mixed with an equal volume of isopropanol (Chem‐labco, Iran; Cat. No A9625), incubated on ice for 20 min, and centrifuged. The RNA pellet was washed with 75% ethanol (Merck, Germany), air‐dried, and dissolved in 40 μL of DEPC‐treated water (Sinaclon, Iran; Cat. No CH8141). RNA was incubated at 55°C for 5 min to ensure complete dissolution.

#### 2.2.2 cDNA Synthesis

First‐strand cDNA synthesis was performed using the RevertAid First Strand cDNA Synthesis Kit (Addbio Inc., Korea; Cat. No. 2102C). The reaction mixture included 5× Reaction Buffer, dNTP mix, oligo dT20, random hexamer, enzyme mix, and DEPC water. After the addition of 5 μL of total RNA, the mixture was incubated at 25°C for 10 min (priming), 50°C for 60 min (reverse transcription), and 80°C for 5 min (enzyme inactivation) using a thermal cycler.

#### 2.2.3 Quantitative Real‐Time PCR

Gene and miRNA expression levels were quantified using the StepOne Real‐Time PCR System with RealQ Plus 2× Green High Rox Master Mix (Ampliqon, Denmark; Cat. No A325402). Each 10 μL reaction contained 5 μL of master mix, 4 μL of RNase‐free water, 0.5 μL each of forward and reverse primers, and 1 μL of cDNA. The thermal cycling conditions were: initial denaturation at 95°C for 3 min, followed by 30 cycles of 95°C for 20 s, 60°C for 20 s, and 72°C for 20 s, with a final extension at 72°C for 10 min.

Primers were designed using Allele ID v7.8 and NCBI Primer‐BLAST. Gene expression levels were normalized to *β‐Actin* for mRNA and *U6* for miRNA. The relative expression was calculated using the 2^−ΔΔCt method. The expression of *miR‐125b, miR‐132*, and the following genes was analyzed: *Il‐1β, Tnf‐α, Nf‐ĸB, P53, Bax, Caspase‐3, Bcl‐2, A20* (*Tnf‐αip3*), *Vegf, Keap1, Nrf2, Sod, Cat, Gpx*, and *Gr* (Chen et al. 2023) (Table 1).

**TABLE 1:** Primer sequences used for real‐time PCR analysis.

Gene	Sequence forward (5′–3′)	Sequence reverse (5′–3′)	Product size (bp)
*Il‐1β*	GACAGTGATGAGAATGACCTGTT	CCCAGGTCAAAGGTTTGGAA	80
*Tnf‐α*	GCCTCCCTCTCATCAGTTCTAT	CACTTGGTGGTTTGCTACGA	104
*Nf‐ĸB*	TCACCAGAAATACCACTGTCAA	ATGGGCCTTCACACACATAG	100
*P53*	AAATGCAGGAGACTTGAGAAA	GCCACAAGTATCTGAAATGGA	99
*Bax*	AGCAAACTGGTGCTCAAGGC	CCACAAAGATGGTCACTGTC	108
*Caspase‐3*	TGACTGGAAAGCCGAAACTC	AGCCTCCACCGGTATCTTCT	122
*Bcl‐2*	GTGGTGGAGGAACTCTTCAG	GTTCCACAAAGGCATCCCAG	245
*A20*	TTTGAGCAATATGCGGAAAGC	AGTTGTCCCATTCGTCATTCC	480 (Qin et al. 2006)
*Vegf*	CCTGGTGGACATCTTCCAGGAGTA	CTCACCGCCTTGGCTTGTCACA	479 (Schmitz et al. 2006)
*Nrf2*	CTGAACTCCTGGACGGGACTA	CGGTGGGTCTCCGTAAATGG	350 (Shanmugam et al. 2019)
*Keap1*	TGCCCCTGTGGTCAAAGTG	GGTTCGGTTACCGTCCTGC	350 (Shanmugam et al. 2019)
*Cat*	AGCTGATCACAGTTCGTGA	ATGGCATCCTGATGAAGA	111
*Gpx*	CCACCGTGTATGCCTTCTC	GGGACGCGACATTCTCAAT	102
*Gr*	CGGAATTCATGCACGATCAC	GCTTGATGACATGCCAACTG	75
*Sod*	CGGATGAAGAGAGGCA	TGTACGGCCAATGATGGA	125
*β‐Actin*	TCCTGACCCTGAAGTACCC	CACACGCAGCTCATTGTAGA	98
*miR‐132*	TCGCTAACCGTGGCTTTCGATT	TTCCGUGGCUUUCGTUUGUUTC	94
*miR‐125b*	TCGCTTCCCTGAGACCCTTTAAC	UCCCUGTGTCCCUUUTTCCUGUGT	88
*U6*	GCTTCGGCAGCACATATACTA	CGAATTTGCGTGTCATCCTTG	92

### 2.3 Histology

The right ovaries were isolated for tissue processing. The ovaries were fixed in 10% buffered formalin (Merck company, Germany) for up to 24 h at 4°C. For dehydration, the samples were placed in varying concentrations of ethanol (70%–100%) (Merck company, Germany). For clarification, ethanol tissue was replaced with the organic solvent xylene (Merck, Germany). To create a paraffin mold, the clarified tissue was dipped in molten paraffin (temperature: 52°C–60°C), and placed in an oven. The samples were embedded in paraffin wax blocks according to the conventional histological method (paraffin). Subsequently, serial sections with a thickness of 5 μm were cut using a microtome. Tissue sections were deparaffinized in xylene and then rehydrated in a series of graded ethanol baths (100%, 95%, and 70%) and water. Tissue sections were stained with hematoxylin–eosin (H&E) (Merck company, Germany) and examined under a light microscope (Nikon, Japan). Ovarian follicles were classified according to their developmental stage: primordial follicles exhibited a single layer of flattened granulosa cells surrounding the oocyte; primary follicles consisted of a single cuboidal granulosa cell layer; secondary follicles displayed two or more granulosa cell layers accompanied by a theca layer; and preovulatory follicles were characterized by a fully developed antrum and a distinct theca cell layer. Additionally, atretic follicles—showing signs of degeneration such as pyknotic nuclei, fragmented granulosa cells, or a collapsed structure (Ataabadi et al. 2023; Jomova et al. 2025). A digital camera from Microbin (Microteb company, Iran) photographed the histological sample.

### 2.4 Statistical Analysis

Statistical tests were performed using the statistical package for the social sciences (SPSS version 24.0, SPSS Inc., Chicago, IL, USA). Data are reported as the means ± standard deviation (SD). Multiple group comparisons were performed using the one‐way analysis of variance (ANOVA) test on GraphPad Prism 9.0 (GraphPad Software Inc., San Diego, USA), followed by a post hoc Dunnett test. The level of significance was set at *p* ≤ 0.05.

## 3 Results

### 3.1 Level of mRNA Expression of Pro‐Inflammatory Cytokines (*Il‐1β, Tnf‐α*)

**FIGURE 1:**
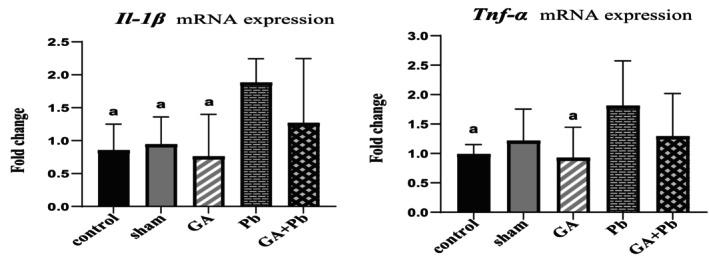
The relative mRNA expression of *Il‐1β* and *Tnf‐α* genes of ovaries in groups. Bar represented by mean ± SD, and *p* ≤ 0.05 was considered significant. GA, gallic acid. Pb, lead acetate. ^a^versus Pb group.

As depicted in Figure 1, the mRNA expression of *Il‐1β* in the Pb group was significantly higher than in the GA, control, and sham groups (*p* = 0.006, *p* = 0.013, and *p* = 0.024, respectively).

The mRNA expression of *Tnf‐α* in the Pb group was also elevated compared to all other groups, with significant differences observed in comparison to the GA and control groups (*p* = 0.028 and *p* = 0.045, respectively). In the (GA+Pb) group, the administration of GA 1 h before Pb reduced the mRNA expression of inflammatory cytokines compared to the Pb group (non‐significant).

### 3.2 Level of mRNA Expression of Apoptotic Genes (*Nf‐ĸB, P53, Bax, Caspase‐3*)

**FIGURE 2:**
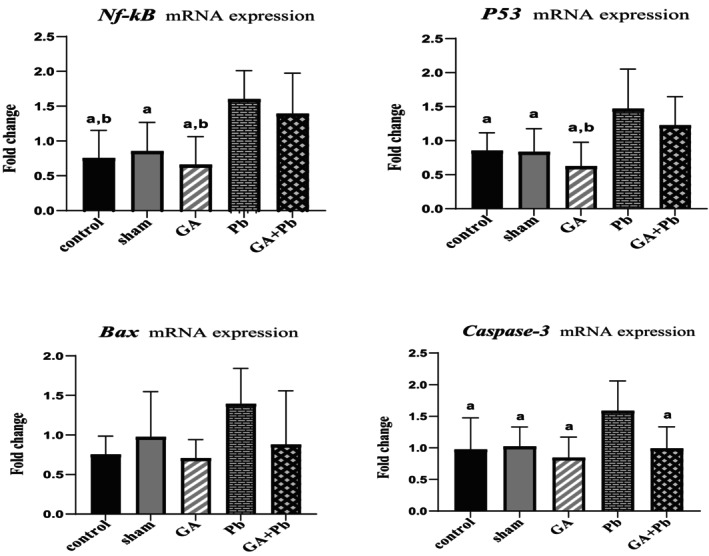
The relative mRNA expression of *Nf‐ĸB, P53, Bax*, and *Caspase‐3* genes of ovaries in all groups. Bar represented by mean ± SD, and *p* ≤ 0.05 was considered significant. GA, gallic acid. Pb, lead acetate. ^a^versus Pb group. ^b^versus GA+Pb group.

According to Figure 2, the mRNA expression of *Nf‐ĸB* was higher in the Pb group compared to other groups, with statistically significant differences observed in comparison to the GA, control, and sham groups (*p* = 0.002, *p* = 0.005, and *p* = 0.014, respectively). Additionally, the mRNA expression of *Nf‐ĸB* in the (GA+Pb) group was significantly increased compared to the GA and control groups (*p* = 0.016 and *p* = 0.042, respectively).

The mRNA expression of *P53* in the Pb group was also higher than in the other groups, with significant differences from the GA, control, and sham groups (*p* = 0.002, *p* = 0.029, and *p* = 0.024, respectively). Furthermore, the mRNA expression of *P53* in the (GA+Pb) group was significantly higher than in the GA group (*p* = 0.035).

The mRNA expression of *Bax* in the Pb group was higher than in the other groups, although the difference was not statistically significant. However, the comparison with the GA group was close to significance (*p* = 0.060).

The mRNA expression of *Caspase‐3* in the Pb group was significantly higher than in the GA, control, sham, and (GA+Pb) groups (*p* = 0.006, *p* = 0.026, *p* = 0.045, and *p* = 0.032, respectively).

### 3.3 Level of mRNA Expression of Anti‐Apoptotic Genes (*Bcl‐2, Vegf, A20* (*Tnf‐αip3*))

**FIGURE 3:**
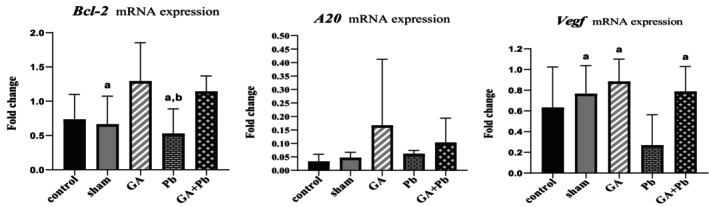
The relative mRNA expression of *Bcl‐2 and A20* genes of ovaries in groups ^a^versus GA group^b^, versus GA+Pb group and the relative mRNA expression of the *Vegf* gene of ovaries in groups ^a^versus Pb group. Bar represented by mean ± SD, and *p* ≤ 0.05 was considered significant. GA, gallic acid. Pb, lead acetate.

The results are shown in Figure 3. The mRNA expression of *Bcl‐2* in the GA group was higher than in the other groups, with significant differences observed when compared to the Pb and sham groups (*p* = 0.009 and *p* = 0.036, respectively). The difference compared to the control group was close to significance (*p* = 0.069). Additionally, the mRNA expression of *Bcl‐2* in the (GA+Pb) group was significantly higher than in the Pb group (*p* = 0.049).

The mRNA expression of *Vegf* in the Pb group was lower than in the other groups, with significant differences compared to the GA, (GA+Pb), and sham groups (*p* = 0.012, *p* = 0.036, and *p* = 0.046, respectively).

The mRNA expression of *A20* (*Tnf‐αip3*) was low in all groups and was close to zero in the control and sham groups. The mRNA expression of *A20* (*Tnf‐αip3*) in the GA group was higher than in the other groups (non‐significant). The mRNA expression of *A20* (*Tnf‐αip3*) in the (GA+Pb) group was higher than in the Pb group (non‐significant).

### 3.4 Level of mRNA Expression of *Keap1* and *Nrf2*

**FIGURE 4:**
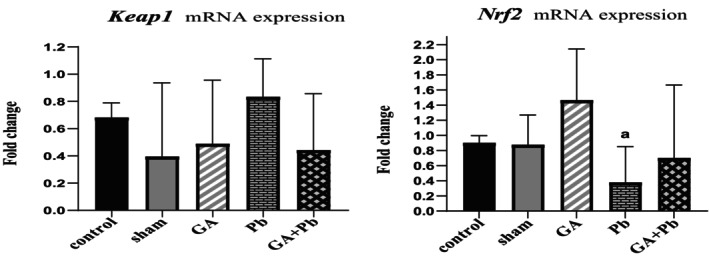
The relative mRNA expression of *Nrf2* and *Keap1* genes of ovaries in the groups. Bar represented by mean ± SD, and *p* ≤ 0.05 was considered significant. GA, gallic acid. Pb, lead acetate. ^a^versus GA group.

The results are shown in Figure 4. The mRNA expression of *Nrf2* in the GA group was higher than in the other groups, with a significant difference observed when compared to the Pb group (*p* = 0.047). The mRNA expression of *Nrf2* in the (GA+Pb) group was higher than in the Pb group (non‐significant).

The mRNA expression of *Keap1* in the Pb group was higher than in the other groups (non‐significant).

### 3.5 Level of mRNA Expression of Antioxidant Enzyme Genes (*Cat, Sod, Gpx, gr*)

**FIGURE 5:**
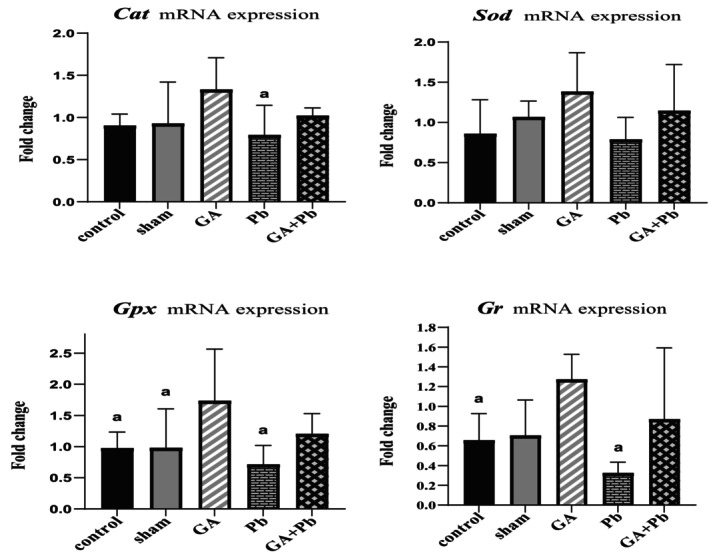
The relative mRNA expression of *Cat*, *Sod*, *Gpx*, and *Gr* genes of ovaries in groups. Bar represented by mean ± SD, and *p* ≤ 0.05 was considered significant. GA, gallic acid. Pb, lead acetate. ^a^versus. GA group.

As illustrated in Figure 5, the mRNA expression level of *Cat* was highest in the GA group compared to all other groups, with a statistically significant increase observed in comparison to the Pb group (*p* = 0.030). In the (GA+Pb) group, *Cat* expression was intermediate between the GA and Pb groups; however, this difference did not reach statistical significance.

The mRNA expression of *Sod* also showed an upward trend in the GA group relative to the other groups, though the increase was not statistically significant. Notably, the difference compared to the Pb group approached significance (*p* = 0.067). In the (GA+Pb) group, *Sod* expression was again between the levels observed in the GA and Pb groups, without a significant difference.

For *Gpx*, mRNA expression in the GA group was significantly elevated compared to the Pb (*p* = 0.003), control (*p* = 0.036), and sham (*p* = 0.037) groups. The expression level in the (GA+Pb) group remained intermediate between GA and Pb, although the differences were not statistically significant.

Similarly, *Gr* (also referred to as *Gsr*) expression was significantly higher in the GA group compared to both the Pb (*p* = 0.002) and control (*p* = 0.045) groups. The difference relative to the sham group approached statistical significance (*p* = 0.069). In the (GA+Pb) group, *Gr* expression was higher than in the Pb group and closer to the GA group, but this increase was not statistically significant.

### 3.6 Level of miRNAs Expression (*miR‐125b, miR‐132*)

**FIGURE 6:**
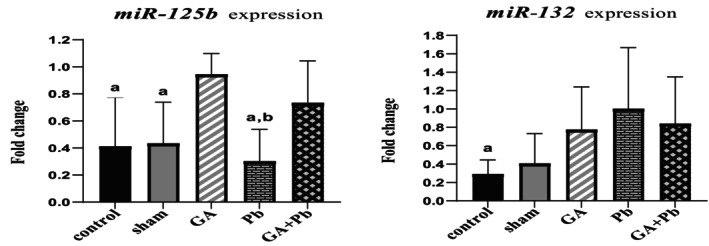
The relative expression of *miR‐125b* in ovaries in groups ^a^versus GA group; ^b^versus GA+Pb group, and the relative expression of *miR‐132* in ovaries in groups ^a^versus Pb group. Bar represented by mean ± SD, and *p* ≤ 0.05 was considered significant. GA, gallic acid. Pb, lead acetate.

The results are shown in Figure 6 ^a,b^.

In the GA group, *miR‐125b* expression was significantly increased compared to the other groups. Notably, the expression levels differed significantly from those observed in the Pb (*p* = 0.002), control (*p* = 0.016), and sham (*p* = 0.015) groups. In contrast, the Pb group exhibited the lowest levels of miR‐125b expression among all groups, with a statistically significant reduction compared to the (GA+Pb) group (*p* = 0.045).

Regarding *miR‐132*, its expression was highest in the Pb group compared to all other groups. This increase was statistically significant relative to the control group (*p* = 0.038). In the GA and (GA+Pb) groups, miR‐132 expression levels were comparable and moderately elevated compared to the control and sham groups; however, these differences were not statistically significant.

### 3.7 The Results of Histology

**FIGURE 7:**
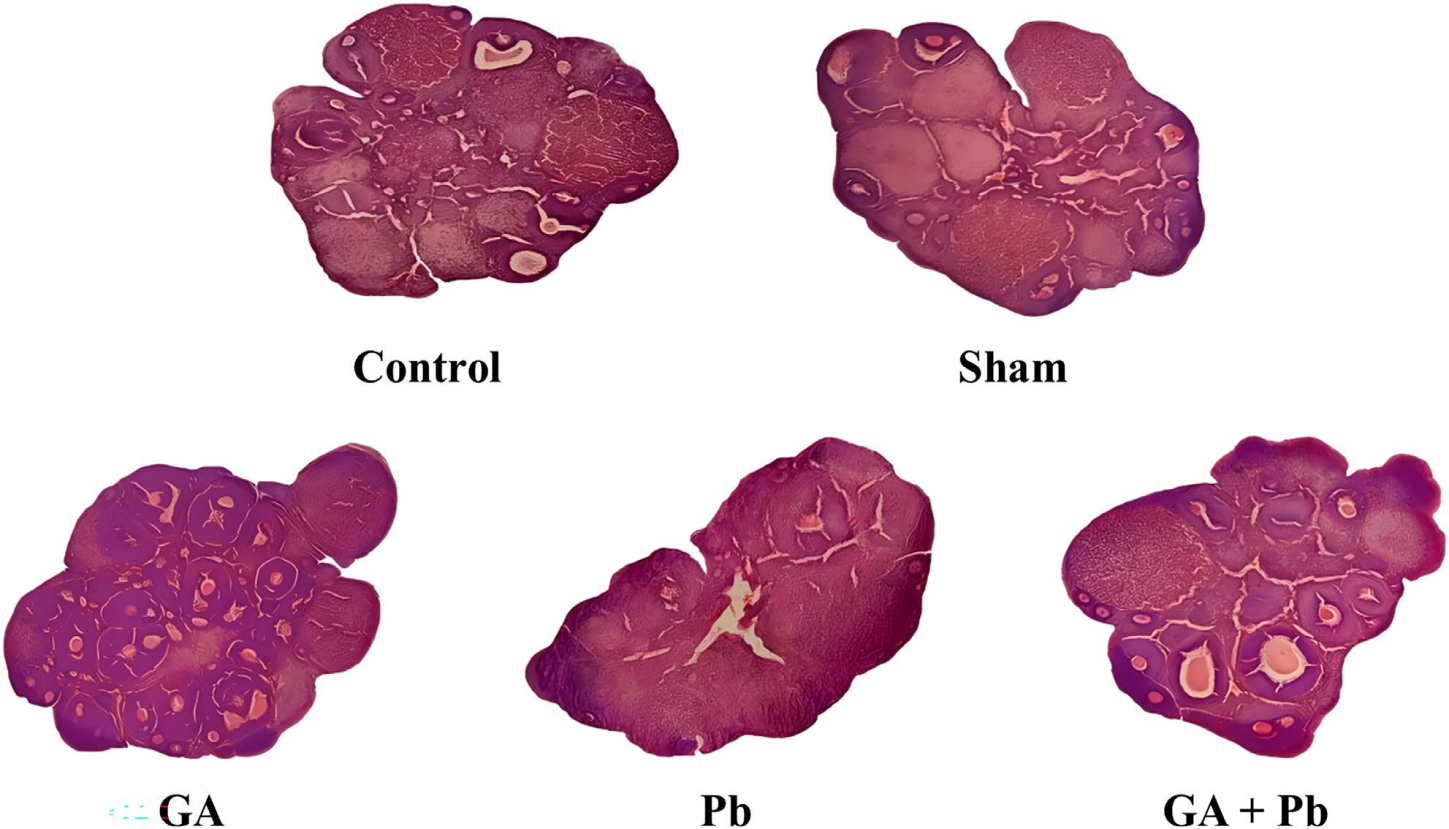
The ovarian tissue of control, sham, GA, Pb, and (GA+Pb) groups. GA, gallic acid. Pb, lead acetate. The tissue structure of the ovarian tissue in the PB group showed destruction and reduced folliculogenesis compared to other groups. Gallic acid alleviated this destruction and increased the number of primordial, primary, secondary, and preovulatory follicles. Pb increased the number of atretic foliicles.

**FIGURE 8:**
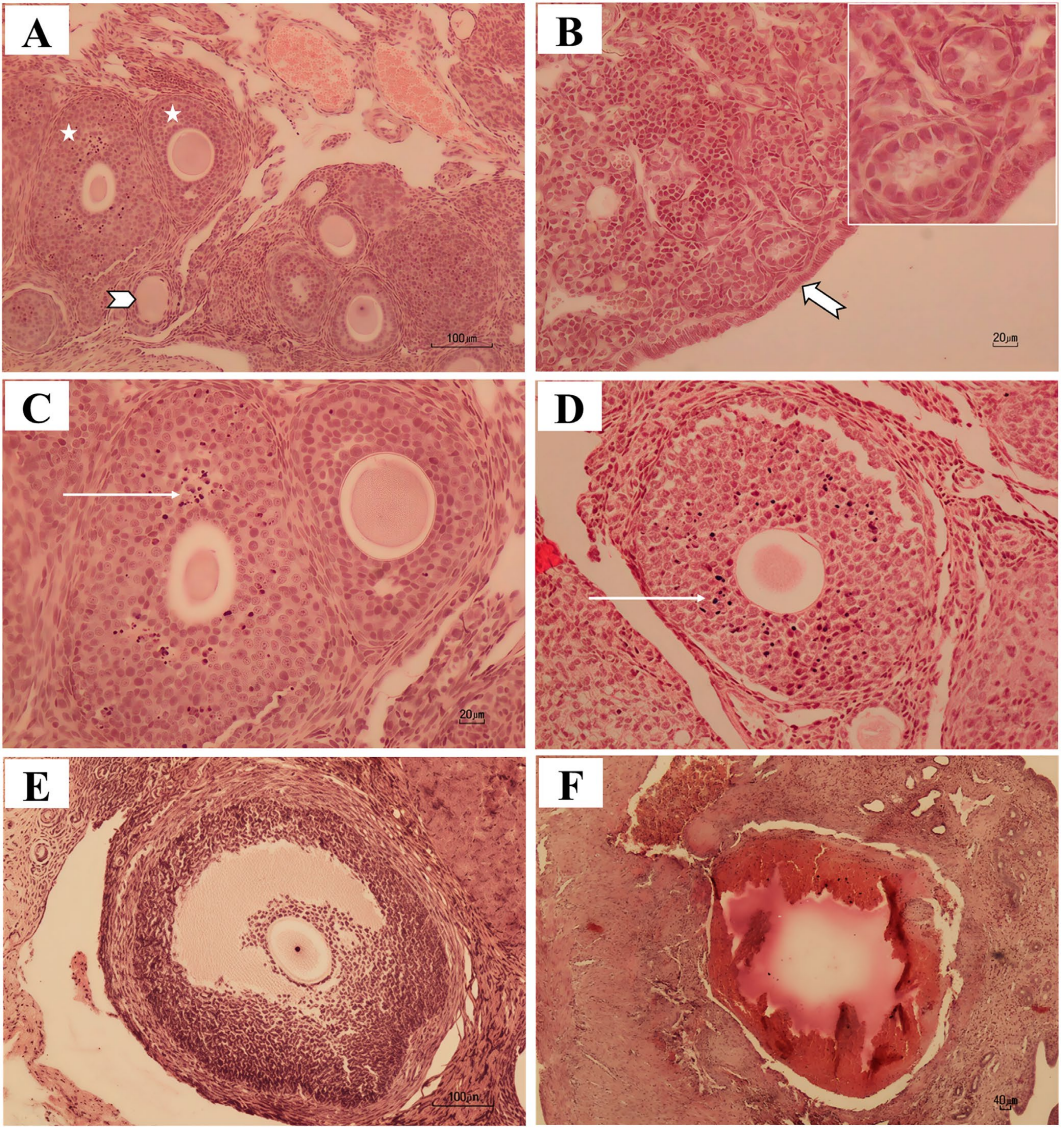
Photomicrographs of different follicles of mice ovarian sections, hematoxylin and eosin staining. (A) Primordial follicle can be found by arrowhead, and secondary follicles by star; (B) Ovarian cortex filled with primordial and primary unilaminar follicles, also the high magnification of these follicles is shown in the upper corner of the photograph, the germinal epithelium shown by arrow; (C, D) Necrotic cells in the secondary follicles of the PB group are shown by arrow; (E) Preovulatorty follicle; (F) Atretic follicle. The scale bars of photographs B, C, and D are 20 μm, in A and E are 100 μm, and in F is 40 μm.

The microscopic images of ovarian sections are presented in Figures 7 and 8. It was observed that the number of follicles differed between the groups. The number of primordial, primary, secondary, and preovulatory follicles was significantly decreased in the PB group compared with all other groups (*p* < 0.5). However, in the GA+Pb group, the number of primordial, primary, secondary, and preovulatory follicles increased in comparison to the PB group, but it was still significantly less than the control, sham, and GA groups (*p* < 0.05). In this group, the number of secondary follicles was similar to the control, sham, and GA groups (*p* > 0.05).

The number of atretic follicles was significantly higher in the PB group compared with all other groups (*p* < 0.5). But in the GA + Pb group, the number of atretic follicles decreased when compared to the PB group, but it did not reach the control, sham, and GA group (*p* < 0.05) (Table 2).

**TABLE 2:** Percentage of ovarian follicles in the study groups.

Groups	Primordial follicles	Primary follicles	Secondary follicles	Preovulatory follicles	Atretic fpllicles
Control	32.17 ± 3.28	29.27 ± 4.06	21.17 ± 2.53	16.00 ± 4.64	1.39 ± 1.43
Sham	34.21 ± 3.82	28.43 ± 3.54	21.65 ± 3.18	14.04 ± 3.16	1.67 ± 2.18
Pb	24.79 ± 2.41^a^	13 ± 2.1^a^	9.38 ± 1.05^a^	5.37 ± 1.43^a^	47.46 ± 4.66^a^
GA	34.53 ± 3.74	27.52 ± 5.84	20.62 ± 3.42	15.84 ± 2.66	1.49 ± 1.42
GA+Pb	28.31 ± 3.12^b^	21.65 ± 4.33^b^	18.94 ± 3.58	9.32 ± 1.38^b^	21.78 ± 2.55^b^

*Note:* Data are shown as mean ± SD. The mean numbers of primordial, primary, secondary, preovulatory, and atretic follicles in the experimental groups. “a” shows significant differences among the Pb group with all other groups. “b” shows significant differences among the GA+Pb group with all other groups (*p* < 0.05).

### 3.8 The Weight of Mice

**FIGURE 9:**
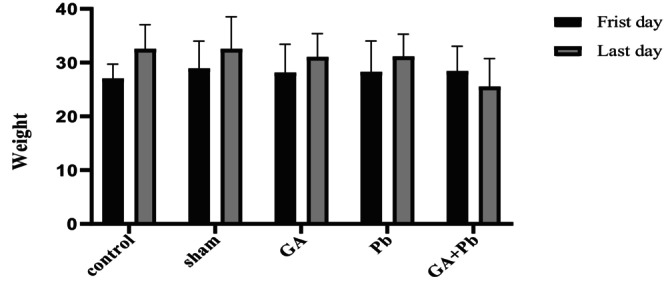
The average weight of mice in groups at the beginning and the end of the experiment (grams). GA, gallic acid. Pb, lead acetate.

Mice were weighed before the initiation of treatments and again at the end of the 35‐day experimental period, just before sacrifice. As shown in Figure 9, the average body weight increased in the control, sham, GA, and Pb groups. In contrast, the GA+Pb group, which received both treatments with a one‐hour interval, exhibited a decrease in body weight by the end of the study.

## 4 Discussion (All Content)

This study elucidates the protective effects of gallic acid (GA) against lead acetate (Pb)‐induced oxidative stress and inflammation in murine ovarian tissue. Pb exposure is known to disrupt reproductive function by inducing oxidative stress, inflammation, and mitochondrial dysfunction, leading to follicular atresia and impaired angiogenesis. Our findings corroborate these effects, demonstrating significant alterations in gene expression associated with oxidative stress, inflammation, and apoptosis in Pb‐exposed ovaries.

Oxidative stress and inflammation are associated with reproductive system disorders and lead to anovulation, PCOS (Polycystic ovary syndrome), implantation failure, and infertility (Sanaz Alaee et al. 2020). Lead acetate (Pb) induces oxidative stress, inflammation, and multigenerational tissue alterations in ovaries (Trujillo‐Vazquez et al. 2023). Pb disrupts the balance between mitochondrial fusion and fission, resulting in mitochondrial dysfunction (Yang et al. 2025). Pb accumulates in the ovary, disrupts the function of reproductive hormones, and has a detrimental effect on the follicle (Qu et al. 2021). In our study, gene expression at the mRNA level also indicated the toxic impact of Pb on the ovary. Pb induced inflammation (*Il‐1β, Tnf‐α*), which led to an increase in the transcription factor *Nf‐ĸB*. Subsequently, *Nf‐ĸB* was freed from its blockage in the cytoplasm and migrated to the nucleus. This led to an increase in *P53*, which could repair damaged cells. However, the damage was probably significant, so the ovaries increased the expression of apoptotic genes (*Bax, Caspase‐3*) and decreased the expression of anti‐apoptotic genes (*Bcl‐2*) to maintain balance and prevent the proliferation of damaged cells. The *Tnf‐α/Nf‐κB/P53* axis is identified as a significant factor in cell death (apoptosis) (Anilkumar and Wright‐Jin 2024).

In our study, the mRNA expression of inflammatory and apoptotic markers in the gallic acid (GA) group was lower compared to the other groups. GA, as a beneficial polyphenol, did not induce toxicity and inflammation; therefore, the body did not need to remove any cells (Tsiogkas et al. 2023). In the (GA+Pb) group, GA was insufficient to halt the toxic effect of Pb on apoptosis through *Nf‐ĸB* and *P53* genes. However, GA reduced the toxic impact of Pb on apoptosis by altering the mRNA expression of *Bcl‐2*, *Bax*, and *Caspase‐3* genes. The protective effect of natural antioxidants has also been confirmed in other studies. GA decreased the expression of *Nf‐κB*, *Bax*, and *Caspase‐3* in the kidney of rats treated with cisplatin, while simultaneously increasing the expression of *Bcl‐2* (Saif‐Elnasr et al. 2023). GA reduced inflammation and apoptosis in the colon of mice treated with trinitrobenzene sulfonic acid (Zhu et al. 2019).

Protein *A20* (*Tnf‐αip3*) is known for its ability to inhibit *Nf‐κB* activity and apoptosis induced by *Tnf*. *A20* is induced by *Nf‐κB* to prevent prolonged activation of *Nf‐κB*, which results in decreased expression of *Tnf‐α* and *Il‐1β* (Prasad and Bao 2019; Wang et al. 2015). The anti‐inflammatory and anti‐apoptotic effects of *A20* have been demonstrated in studies. In mouse liver, heavy metal cadmium (Cd) reduced the expression of *A20* but induced inflammation and increased the phosphorylation of p65 and IκBα, which was associated with the release of *Nf‐κB*. Overexpression of *A20* effectively protected against liver inflammation induced by exposure to Cd (Jia et al. 2023). Natural antioxidants increase the expression of gene *A20*. Vitamin E enhances the expression of *A20* and subsequently decreases inflammation and apoptosis in mice macrophage (Wang et al. 2015). Zinc acts as an antioxidant and increases the expression of *A20* while reducing *Tnf‐α*, *Il‐1β*, and *Nf‐κB* in various organs (Jarosz et al. 2017). The result of our study was also in line with the findings of the mentioned studies. The expression of *A20* in the Pb group was low, while in the GA group it was higher than in the others. Overall, the expression of *A20* was very low across all groups, and in the control and sham groups, it was close to zero. An unknown factor may have inhibited the mRNA expression of *A20* in the ovaries.

In our study, the mRNA expression of *Vegf*, known as an angiogenic gene, was highest in the GA group and lowest in the Pb group. The mRNA expression of *Vegf* in the (GA+Pb) group was greater than in the Pb group. This indicates that GA effectively counteracts the inhibitory effect of Pb on angiogenesis. The data from some studies were similar to our results. Cadmium is a heavy metal that reduces the expression of *Vegf* in trophoblasts of mouse and human placentas (Xiong et al. 2021). Resveratrol is a polyphenol that increases the expression of *Vegf* to promote angiogenesis following duck ovary transplantation (Qin et al. 2023). Pb in the pituitary and ovary of rats caused follicular death and decreased the expression of growth factors, including *Vegf*. Dietary treatment with edible bird's nest (EBN) eliminated the destructive effects of Pb and increased *Vegf* expression. EBN contains beneficial substances, including antioxidants (Albishtue et al. 2018). In contrast to our study, a review article has stated that Pb induces hypoxia, and the factors induced by hypoxia, in turn, stimulate the expression of *Vegf* (Josko and Mazurek 2004).

A study on human lungs found that heavy metals, especially Pb, reduce the expression of *Nrf2*, antioxidant enzymes, and GSH levels (Gogoi et al. 2019). Pb interferes with the function of antioxidant enzymes; for example, it competes with zinc in *Sod* and prevents its function, leading to oxidative stress (Xiao and Lai 2025). A study reported that *Keap1* is a negative regulator of *Nrf2, as* the expression of *Nrf2* in *Keap1*‐knockdown mice was significantly higher than that in wild‐type mice (Oishi et al. 2020). Studies have revealed that polyphenols potentially mitigate toxicity caused by heavy metals. GA increased the expression of *Nrf2*, *Sod*, *Cat*, and *GSH* while reducing *Nf‐κB* and *Caspase‐3* in the kidney of rats treated with uranyl acetate (Ragab et al. 2025). GA reduced oxidative damage caused by Pb in the blood, liver, and kidney of rats (Reckziegel et al. 2016). In our study, GA caused a decrease in the mRNA expression of *Keap1* (non‐significant), while increasing the mRNA expression of *Nrf2*, *Cat, Sod, Gpx*, and *Gr* in comparison to other groups. As expected, Pb reduced the antioxidant capacity of the ovary at the mRNA level. The mRNA expression of *Nrf2*, *Keap1*, and antioxidant enzymes in the (GA+Pb) group was similar to that in the GA group. This indicates the positive effect of polyphenols in preventing the decrease in antioxidant capacity caused by toxic substances.

The practical effect of mRNA expression of the examined genes was confirmed by microscopic observations. The toxic effect of Pb stimulated inflammation and subsequently the death of ovarian cells and a reduction in angiogenesis. GA stimulated the growth of follicles and blood vessels. In the (GA+Pb) group, GA fought against the oxidation caused by Pb, and the ovaries were histologically different from those in the Pb group. Our histological results are confirmed by other studies. Mice that were exposed to low‐dose Pb for a long time had altered ovarian tissue structure and an increased number of atretic follicles (Trujillo‐Vazquez et al. 2023). A study on rats demonstrated that metals (Pb, Hg, Mn, and Al) caused degeneration of ovarian cortex and induced apoptosis in follicles (Eddie‐Amadi et al. 2023). GA prevented the deleterious impacts of cadmium on the histopathological structure of the ovaries in rats (Rotimi et al. 2021).

It was observed that the expression of *miR‐125b* was highest in the GA group and lowest in the Pb group. The expression of *miR‐125b* in the (GA+Pb) group was similar to the GA group. Previous findings indicated that in the GA group, the mRNA expression of antioxidant and anti‐apoptotic genes was elevated, while the mRNA expression of inflammatory and apoptotic markers was reduced. Therefore, it can be concluded that *miR‐125b* increases the expression of antioxidant and anti‐apoptotic genes in the mice ovary, while decreasing the expression of inflammatory and apoptotic genes. The results of some studies were consistent with ours. Research on the silver carp spleen demonstrated that *miR‐125b* caused an increase in the expression of antioxidant enzymes and a decrease in the expression of inflammatory and apoptotic genes (Ma et al. 2019). Some studies contradict our findings. *MiR‐125b* in human DLBCL (diffuse large B‐cell lymphoma) caused a decrease in *A20* (*Tnf‐αip3*) expression and an increase in the expression of *Tnf‐α* and *Nf‐κB* (Kim et al. 2012). *MiR‐125b* induced an increase in *Bax* expression and a decrease in *Bcl‐2* expression in the ovaries of rats with PCOS (Polycystic ovary syndrome). This led to apoptosis in the granulosa cells (Xuan et al. 2024).

The expression of *miR‐132* in the Pb group was higher than in the others, and in the GA group, it was higher than in the control and sham groups (non‐significant). Interestingly, Pb and GA, alone and together, increased the expression of miR‐132 compared to the control and sham groups. In the Pb group, there was an inverse relationship between *miR‐125b* and *miR‐132*. However, in the GA group, the expression of *miR‐125b* and *miR‐132* was elevated. These findings were consistent with similar studies. Cadmium and arsenic, as heavy metals, increased the expression of *miR‐132* in the kidney cortex of mice and humans, leading to inflammation, cell death, and serious damage (Pellegrini et al. 2016). In a study on the mouse brain, GA enhanced the expression of *miR‐132*, resulting in a reduction of inflammatory cytokine levels (Abdullah et al. 2019). Some studies contradict our results. In the mouse brain, aluminum increased the expression of inflammatory‐apoptotic genes by elevating *miR‐125b* and decreasing *miR‐132*, while it reduced the expression of antioxidant and anti‐apoptotic genes. Administration of phenolic extract altered all the outcomes (El Gizawy and Boshra 2023).

The variability in the expression of miRNAs within identical treatments indicates that each miRNA does not have a fixed role. MiRNAs contribute to the maintenance of homeostasis by activating or inhibiting gene expression in response to the current cellular conditions and the presence of other miRNAs and mRNAs. Differences in tissue type and species also play a role in this process.

In our study, mice that received treatments twice daily via gavage exhibited a lower final weight compared to the first day, whereas other groups gained weight. The stress induced by gavage may have contributed to weight loss. Similar findings have been reported in other studies. Multiple daily gavage resulted in increased stress and elevated plasma corticosterone levels in mice and rats (Larcombe et al. 2019). Administration of normal saline via gavage twice daily resulted in increased stress, elevated metabolic rate, and a reduction in body fat and overall weight in comparison to once‐daily gavage administration and the control group without gavage (de Meijer et al. 2010).

## 5 Conclusion

Our findings demonstrate that gallic acid (75 mg/kg), a potent polyphenolic compound, exerts antioxidant, anti‐inflammatory, and anti‐apoptotic effects in the ovaries of mice, effectively counteracting the ovarian damage induced by lead acetate (30 mg/kg). Considering the ubiquitous environmental contamination by heavy metals—frequently introduced into biological systems through polluted food sources, drinking water, and air—dietary supplementation with polyphenolic compounds represents a promising approach for mitigating the toxicological impact of such exposures. These bioactive molecules play a critical role in preserving female reproductive health by maintaining ovarian structure and mitigating heavy metal‐induced damage to both the cortical and medullary regions of the ovary. Therefore, polyphenolic compounds hold therapeutic potential for enhancing fertility and safeguarding reproductive function in environments burdened with toxicants.

## Author Contributions

**Fatemeh Zahedi:** conceptualization (equal), funding acquisition (equal), methodology (equal), writing – original draft (equal). **Rasoul Kowsar:** conceptualization (lead), data curation (equal), supervision (lead), writing – review and editing (equal). **Zahra Khodabandeh:** conceptualization (equal), funding acquisition (equal), investigation (equal), methodology (equal), writing – review and editing (equal). **Mahintaj Dara:** conceptualization (equal), investigation (equal), methodology (equal), writing – review and editing (equal). **Sanaz Alaee:** conceptualization (equal), investigation (equal), supervision (equal), writing – review and editing (equal).

## Ethics Statement

The experimental protocol of the animal study was approved by the ethical committee of Shiraz University of Medical Sciences, Shiraz, Iran (IR.SUMS.REC.1400.199).

## Conflicts of Interest

The authors declare no conflicts of interest.

## Data Availability

The data are available upon reasonable request from the corresponding author.
